# Functional and Radiological Outcomes Following Simultaneous Bilateral Total Hip Arthroplasty: Analysis of a Retrospective Series

**DOI:** 10.7759/cureus.46361

**Published:** 2023-10-02

**Authors:** Santosh Bindumadhavan, Anirudh Sharma, Vijay Killampalli

**Affiliations:** 1 Trauma and Orthopaedics, North West Anglia NHS Foundation Trust, Huntingdon, GBR; 2 Trauma and Orthopaedics, Worcestershire Royal Hospital, Worcester, GBR

**Keywords:** total hip replacement, sequentially staged bilateral thr, simultaneous bilateral thr, oxford hip score, global hip offset, limb length discrepancy

## Abstract

Introduction

The prevalence of bilateral hip arthritis continues to rise. With the dramatic change in the practice of modern-day arthroplasty with standard operating protocols and guidelines in place to reduce the incidence of surgical site infection and peri-operative thromboembolic events, simultaneous bilateral total hip replacement (THR) has been considered a viable option to reduce morbidity. The efficacy of simultaneous bilateral THR with regard to patient outcomes and complications has been debated. The aim of this study was to assess and compare the functional outcomes, radiological outcomes, and complications following bilateral simultaneous THR with the existing literature.

Methods

We conducted a retrospective study of 28 patients who underwent simultaneous bilateral THR by a single surgeon at a district general hospital in the United Kingdom between 2010 and 2019. All the patients in the study were operated on via the posterior approach to the hip and received uncemented implants. Data on blood loss and hospital stay were collected from the hospital records. The radiological outcomes were studied from the post-operative radiographs. The patient-reported outcomes were measured via a telephone session at an average follow-up of 7.8 years.

Results

Our results noted a statistically significant drop in haemoglobin after the procedure from a mean of 13.5 g/dl to 9.05 g/dl (t: -15.84, p < 0.00001). The average blood loss was 643 ml +/- 330 ml (200-1850 ml). Nine patients (32.1%) required blood transfusions and a total of 21 units were transfused. The mean duration of stay in the hospital was 6.7 days (three to 20 days). There were no intra-operative/immediate/early post-operative complications. The revision rate was 1.7%, as one patient had a revision of the femoral component following a peri-prosthetic fracture. The mean visual analogue scale pain score was 1.51 +/- 0.58 (1-3). The mean Oxford Hip Score improved from 19.5 (12-28) pre-operatively to 44.3 (37-48) post-operatively at the time of the study (t: -21.88945; p < 0.00001), with the difference being statistically significant using a paired t-test. From the series, 14 (50%) patients were found to have limb length discrepancies. The mean limb length discrepancy was found to be 2.3 mm (0-16 mm). In 13 of the 28 patients (46.4%), the global hip offset was equal on both sides. In two patients, the difference in the global hip offset was more than 10 mm.

Conclusion

We reported good patient-reported functional outcomes with simultaneous bilateral THR with a low complication rate. Despite the lack of opportunity to template the second hip, the limb length and global hip offset can be restored after a simultaneous bilateral THR.

## Introduction

Osteoarthritis (OA) of the hip is a debilitating degenerative joint disorder that impairs patient mobility and leads to increased utility of healthcare services. The burden of OA has rapidly increased over the last few decades and continues to rise due to increased lifespan. Approximately 10 million people in the United Kingdom suffer from arthritis [1]. About 11 in every 100 people over the age of 45 years have OA of the hip and the prevalence rate of adult hip OA globally is around 18.70 per 100,000 individuals [1-3]. Total hip replacement (THR) is the most effective surgical treatment for painful disabling hip arthritis [4]. With advances in surgical and post-operative care, the patient’s quality of life and average lifespan have significantly improved. Good outcomes in patients with hip arthritis and access to surgery have led to an increase in the number of patients undergoing the procedure [5].

Hip OA can affect both hips either sequentially or simultaneously. Most patients who develop arthritis on one side ultimately progress to arthritis on the other side of the hip, with the estimated prevalence of bilateral hip OA ranging between 42% and 50% [6]. Of these, 15-25% of patients end up with bilateral hip OA that warrants surgery for both hips [7].

The practice of performing a simultaneous bilateral THR was first introduced by Jaffe and Charnley in 1971 [8]. Since then, multiple studies have been conducted to assess the risks and benefits of a simultaneous versus sequentially staged bilateral THR [7,9-15]. The practice of simultaneous bilateral THR was shown in earlier studies to be associated with an increase in complications such as deep vein thrombosis (DVT), prosthetic joint infection (PJI), heterotrophic ossification (HO), and decreased gait stability [9-11]. Recent literature has shown equivalent rates of mortality and PJI when compared to staged procedures, but increased rates of pulmonary embolism (PE) [16]. The advocates of simultaneous bilateral THR argue that it is cost-effective by reducing the overall length of hospital stay and anaesthetic and surgical time without any significant change in functional outcome as compared to a staged procedure [12-14].

While previous literature has focussed mainly on the parameters mentioned above, there has been little mention of the accuracy of component placement, restoration of offset, and limb length discrepancy (LLD) when undertaking a simultaneous bilateral THR. Although pre-operative templating is a useful tool, ideally a radiograph of the opposite operated side would be needed to minimise limb length inequality while undertaking the procedure on the second side. Similarly, global hip offset may vary between the two sides based on differences in acetabular reaming and medialisation. We hypothesised that as a radiograph of the first operated side is unavailable prior to performing the second side in a simultaneous bilateral THR, there is a potential for LLD and suboptimal global hip offset restoration.

We conducted a retrospective cohort study of patients undergoing simultaneous bilateral THR at our institution to assess the functional outcomes, radiological outcomes, and complications following bilateral simultaneous THR and compare it with the published literature.

## Materials and methods

We conducted a retrospective cohort study on a series of patients who underwent a simultaneous bilateral THR performed by a single specialist surgeon at a district general hospital in the United Kingdom between 2010 and 2019. All patients underwent the procedure on the day of their admission, under a single anaesthetic episode. Both sides were operated by the same surgeon, and in a theatre setup equipped with laminar airflow. Each patient received one dose of prophylactic antibiotic 30 minutes prior to the first incision. The procedure in all patients was performed via a standard posterior approach to the hip. After the closure of the first hip, the patient was re-positioned and the opposite hip was prepared and draped. The surgeon re-scrubbed prior to the second procedure. All patients received mechanical and pharmacological prophylaxis for venous thromboembolism, as per institutional guidelines. Clinical records, including clinic letters, operative notes, and radiographs, were used to collect data for the study.

Pre-operative factors studied included age, gender, indication for surgery, American Society of Anesthesiologists (ASA) grading, and body mass index (BMI). The pre-operative Oxford Hip Score (OHS), which is a routinely administered questionnaire in our institution pre-operatively, was noted. The intra-operative blood loss was noted from operative records, and the pre-operative and post-operative haemoglobin levels were noted for all patients. The number of patients who had a blood transfusion and the number of units transfused were noted.

**Figure 1:**
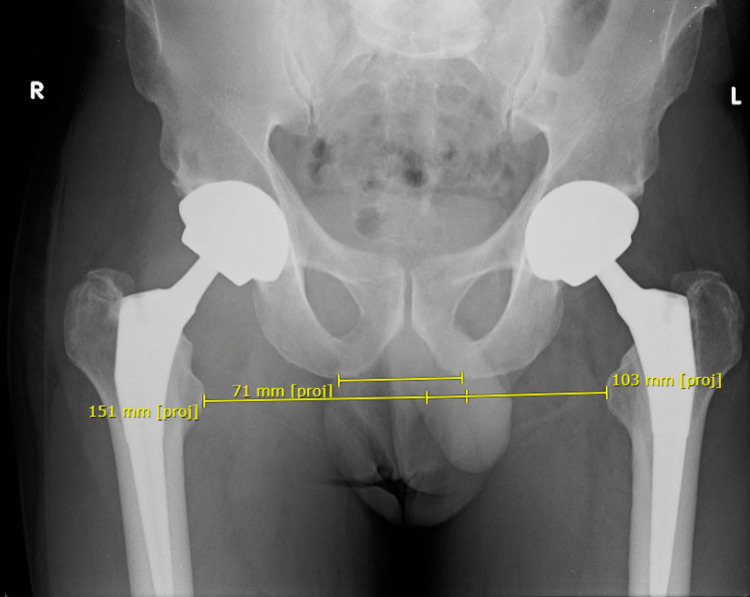
Measurement of limb length discrepancy

**Figure 2:**
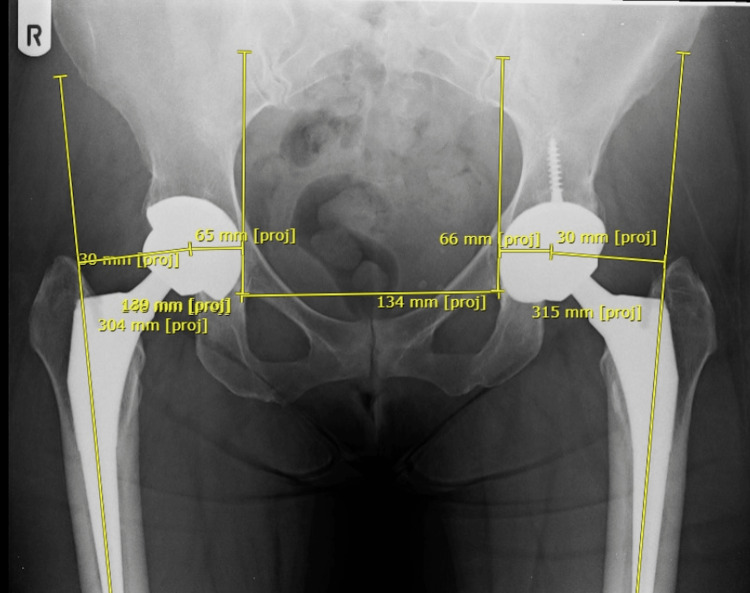
Measurement of global hip offset

Radiological assessment for LLD was performed on the immediate post-operative anteroposterior (AP) view of the pelvis, using digital measurement tools from the institution’s PACS (picture archiving and communication system). The LLD was defined as the difference in the perpendicular distance in millimetres between two lines - the first passing through the lower edges of the ischial tuberosities, and the second passing through the most prominent point of the corresponding lesser trochanter (Figure 1). The global hip offset (GO) was calculated using the same AP radiograph of the pelvis. The GO was defined by the sum of the femoral offset (FO) and the acetabular offset (AO) and was measured for both sides (Figure 2).

Patient-reported outcomes, including the post-operative OHS and visual analogue scale (VAS) for pain, were studied by delivering these questionnaires to all included patients either via post or a telephonic interview. These were assessed for all included patients during the study period from January to April 2023, and the duration since surgery at the time of the present study was noted. Analysis of post-operative complications was done from clinical records, including intra-operative notes, post-operative clinical letters, and serial post-operative radiographs.

## Results

Demographics and pre-operative factors

A total of 28 patients comprising 15 males and 13 females were included in the study. The mean age of the patients in our study was 63 years (36-88 years). The mean ASA grade of the patients was 2 (1-3). The mean BMI was 29.78 (18-41) with the distribution of range shown in Table 1. The indications for bilateral THR have been illustrated in Table 2.

**Table 1:** Distribution of patients based on the body mass index

BMI	No. of patients
<18.5	1
18.5-24.9	3
25.0-29.9	8
30.0-34.9	12
35.0-39.9	3
>40.0	1

**Table 2:** Distribution of patients based on aetiology OA: osteoarthritis.

Aetiology	Total = 28
Primary OA	16
Secondary OA	12
Dysplasia	8
Avascular necrosis	2
Previous trauma	2

Blood loss and transfusion requirements

The average blood loss during the procedure was found to be 643 ml +/- 330 ml (200-1850 ml). The mean pre-operative haemoglobin (Hb) was 13.5 and the mean postoperative Hb was 9.05. There was a statistically significant difference in the pre-operative and post-operative Hb following the procedure (t: -15.84, p < 0.00001) as measured by the dependent t-test. Nine patients (32.1%) required blood transfusions and a total of 21 units were transfused.

Length of hospital stay

The mean duration of stay in the hospital was 6.7 days (three to 20 days). Out of the 28 patients, 26 (92.8%) were discharged within six days. Two patients stayed in the hospital for 16 and 20 days, respectively, due to slow rehabilitation in view of their elderly age.

Complications

There were no intra-operative complications in any patient. None of the patients had any immediate/early post-operative complications. One hip required revision of the femoral component due to a peri-prosthetic fracture sustained after a fall four years following the procedure, resulting in an overall all-cause revision rate of 1.7% in the series. None of the patients developed a DVT, and serial radiographs did not show the development of HO in any patient. No patient developed a PJI.

Radiological outcomes

From the series, 14 (50%) patients were found to have LLD. The mean LLD was found to be 2.3 mm (0-16 mm). Only one patient had an LLD of >10 mm (16 mm). None of the patients with an LLD required any external aids to aid in mobilisation due to limb length changes. In 13 of the 28 patients (46.4%), the GO was equal on both sides. In two patients, the difference in the GO was more than 10 mm.

Patient-reported outcomes

The mean duration since surgery at the time of administering the VAS and OHS questionnaires was 7.8 years (four to 12 years). The mean VAS pain score was 1.51 +/- 0.58 (1-3). The mean Oxford Hip score improved from 19.5 (12-28) pre-operatively to 44.3 (37-48) post-operatively at the time of the study (t: -21.88945; p < 0.00001) with the difference being statistically significant using a paired t-test. Patients with LLD > 10 mm or global offset difference > 10 mm did not have any significant difference in their patient-reported outcomes, compared to the rest of the series (p = 0.34).

The key results of the study have been summarised in Table 3.

**Table 3:** Summary of key results

Parameters	Mean	Standard deviation	Range
Age (years)	63.5	+/-12.6	44-86
Hospital stay(days)	6.7	+/-3.6	3-20
Intra-operative blood loss (ml)	643	+/-330	200-1850
Pre-operative haemoglobin levels (g/dl)	13.5	+/-1.07	11.1-15.1
Post-operative haemoglobin levels (g/dl)	9.05	+/-1.65	4.6-11.5
Limb length discrepancy (mm)	2.3	+/-3.85	0-16
Visual analogue scale pain score	1.5	+/-0.58	1-3
Oxford Hip Score (follow-up)	44.3	+/-2.5	37-48
Oxford Hip Score ( pre-operative)	19.5	+/-5.34	12-28

## Discussion

Our study shows good medium-term patient-reported functional outcomes following simultaneous bilateral THR at a mean follow-up of 7.8 years. Radiological assessment did not show any worse LLD when compared to standard mean values of 3-17 mm after THR described in a literature review [16], thus negating our initial hypothesis. In our series, parameters such as intra-operative blood loss, mean hospital stay, and post-operative complications, such as thromboembolic events, showed an improvement over existing studies, although this should be taken in the context of our series being low volume.

The importance of restoration of limb length and global hip offset in THR is well known. Renkawitz et al. analysed the gait of 60 patients with unilateral THA and noted the restoration of LLD and GO within +/- 5 mm improved hip range of movements and gait kinematics [17]. Restoring limb length and global offset can be a challenge during simultaneous bilateral hip replacements as there is no opportunity to template during the second surgery. There is no defined limit of acceptable LLD in terms of hip function and patient satisfaction, but, there is a general consensus among hip surgeons that most patients can tolerate a discrepancy of <10 mm and between 15% and 20% of those with LLD > 10 mm are symptomatic [18,19]. A previous study by Kim et al. noted that the mean postoperative LLD was significantly lower in the simultaneous bilateral THA group (2.1 mm) compared to the staged group (4.3 mm) [20]. In our study, the mean post-operative LLD was 2.3 mm, which is comparable to the above study. We observed that 50% of the patients (n = 14) in our study had no LLD and the global offset was equal bilaterally in 46.4% (n = 13) of patients.

The average blood loss in our study was 643 ml. As compared to other studies, this falls within the lower range of blood loss described previously during simultaneous bilateral THR, which ranges from 497 ml to 1997 ml [21,22]. We note a statistically significant fall in haemoglobin levels postoperatively in our series and 32.1% (n = 9) of patients needed blood transfusions with a mean of 2.3 units for those transfused. This is comparable to the study of Bhan et al. showing a mean of 2.73 units transfused in their group undergoing simultaneous bilateral THR [23]. Previous studies such as those by Salvati et al. and Alfaro-Adrian et al. suggest that the transfusion requirements are less in staged bilateral THA compared to simultaneous bilateral THR possibly owing to the increased cumulative blood loss in a single setting [7,22].

Some studies [9-11,16] have demonstrated an increased incidence of thromboembolic events following simultaneous bilateral THR. However, a meta-analysis of the data for DVT and pulmonary embolism noted no significant difference between bilateral simultaneous and staged bilateral THR [24]. This could be attributed to advances in hypotensive anaesthesia, cementing technique, and peri-operative thromboprophylaxis. While there was no thromboembolic event noted in our series, this may be due to strict adherence to post-operative mechanical and pharmacologic thromboprophylaxis, and the low numbers in our series.

The patients in our study had ASA grades 1-3. None of the patients in our study were ASA grade 4, as it was a planned elective procedure, and this may explain our lower complication rates. There were no systemic peri-operative complications in our study. Alfaro-Adrian et al. reported a higher incidence of peri-operative complications with patients with ASA grade 3 and 4 in staged as well as simultaneous bilateral THR [7].

In cases of hip arthroplasty, the length of hospital stay may contribute to a significant cost incurred. The length of hospital stay is a good indicator of the efficacy and safety of a procedure. The mean length of hospital stay has substantially reduced over the past two decades owing to the improvement in surgical and hospital care. Eggli et al. noted that the mean hospital stay was five to six days shorter in simultaneous bilateral THR as compared to staged THR [15]. We report a mean hospital stay of 6.7 days with a majority of the patients discharged by day six post-operatively. Based on the available literature, this is considerably less than the overall hospital stay for staged bilateral THR [9,13,16,25,26].

Over a mean follow-up of 7.8 years, only one patient underwent a revision of the femoral component due to a peri-prosthetic fracture. No other intra-operative or post-operative systemic complications were noted in our study. Ritter and Vaughan noted an increase in rates of HO following simultaneous bilateral THR [27], but this has not been seen in our study. No dislocations were noted in our series, which suggests that the overall positioning of the components was not compromised during simultaneous bilateral THR.

Functional outcomes of our series using patient-reported scores (OHS and VAS) suggest good patient outcomes and satisfaction following the procedure. Multiple studies opine that bilateral simultaneous THR has equivalent functional outcomes compared to a staged THR [21-23]. Interestingly, functional outcomes may not truly reflect the technical aspects of the procedure due to patient acceptance or endurance and acclimatisation to an altered lifestyle.

Our study has certain limitations. It is a retrospective study from a single centre with a small sample size. As it is a retrospective study, we cannot define the inclusion criteria predisposing selection bias. We did not assess the costs involved, and it was not a comparative study. However, our radiological analysis of LLD and GO in simultaneous bilateral THR adds evidence to the existing literature on a lesser-studied aspect of this procedure.

## Conclusions

The results from our study suggest good patient-reported functional outcomes with simultaneous bilateral THR, with a low complication rate. Our radiological analysis suggests that despite the lack of opportunity to template the second hip, LLD after a simultaneous bilateral THR is no worse than the mean LLD described in the literature for THR. In those with LLD or differences in global hip offset, functional outcomes are not affected. Blood loss and transfusion requirements remain concerns with simultaneous bilateral THR, but thromboembolic events, HO, and PJI were not seen in our series. Thus, simultaneous bilateral THR remains a safe option for patients with bilateral hip arthritis with factors such as appropriate patient selection, skilled surgical expertise, and robust standard operating protocols.
